# Allegheny County Medical Society

**Published:** 1890-01

**Authors:** 


### ALLEGHENY COUNTY MEDICAL SOCIETY.

### SPECIAL MEETING, NOVEMBER 19th, 1889.

W. F. Knox, M. D., President, in the chair.

#### CASE OF AMPUTATION AT HIP JOINT.

Dr. Murdoch.—This case of amputation at the hip joint I did at the West Penn. Hospital, on the 28th of August—twelve weeks ago. Recovery from an amputation at the hip joint is a very rare occurrence in an adult. The operation has been done a good many times. I have performed it several times myself, and this is the first case of recovery I have had. The case is also interesting because of the disease. I have in my hand the femur of the patient who suffered this amputation. It is from a girl eighteen years of age, from Beaver county, a native of Pennsylvania. When, last February, she suffered some pains in the upper part of the thigh, she applied to Dr. Simpson, of New Brighton, who recognized an osteo-sarcoma, and brought the girl to me at the West Penn. Hospital on the 23d of August. The tumor was then very much enlarged, and I amputated at the hip joint. The disease is, as you see, osteo-sarcoma; it involves chiefly the periosteum, and that is said to be the most malignant type of that disease, more likely to recur than when it attacks the center of the bone. It has been twelve weeks since the amputation, and the girl is now in perfect health. At the time of the operation she was emaciated, could not sleep without large doses of morphia, and since the amputation she has been in comparatively good health, her pain has left, and there has been no return of the disease in the stump. Amputation at the hip joint possesses an exceedingly great mortality. So great is the mortality following primary amputation of the hip joint that of the twelve amputations made in the Crimean war—primary amputations—all died. Previous to our own war, of thirty cases of primary amputations, owing to gunshot injuries, not one survived. The history of primary amputation at the hip joint, in our war, is a little more favorable, but not much; there were nineteen primary amputations, and of these, eleven died from the shock of the operation within a few hours after amputation; five died within forty-eight hours from other causes, and there were only three who recovered, and only one that was known to be alive two years after the amputation and that one case I feel particular interest in, as I was the surgeon who controlled the artery at the time of the amputation. This is known in the history of the war as the Shippens case, and was performed on a Pennsylvania private, a young man by the name of John Kelly, who is now living at Black Lick, in this State, in excellent health. This is the only case on record, I believe, that has lived over two years after primary amputation at the hip joint. Amputations for disease are a little more favorable than for injury, and there are a great many cases of recovery, but still even then it is a very fatal operation. Mr. Lister's abdominal tourniquet has been a great assistance to surgeons in this operation. In the case I report, I was able to control the hemorrhage very effectively by the reliable assistants who assisted at the operation. I will not go into a description of the operation. The girl is now well. I pass this specimen (the tumor and the femur) around among you; it is a beautiful specimen of osteo-sarcoma, one of the finest of which I know.

TWO CASES OF TREPANATION.

DR. MURDOCH.—I desire to report, also, two very remarkable cases at the West Penn Hospital. One I have here; the other is not here. A boy was injured in an explosion at the West Point boiler works on the 14th day of March, at noon, and brought to the West Penn Hospital in the afternoon. When he arrived there, there was a small scalp wound on the left side of his head, and on the right side of the head the hair was burned off and the scalp severely burned. It was dressed as a burn ; the boy was intelligent, answered questions, and seemed to be not very much hurt. In the course of three or four days, this burn on the right side of the head sloughed ; the burn had been so severe as to destroy the scalp circularly, two inches in diameter. In the centre the bone was exposed about the size of a silver half-dollar. The boy became stupid, irritable and disposed to sleep, and began to draw his knees up to his chin ; in three or four days he had lost the use of his left arm and leg ; he had neither motion nor sensation. It was thought by some of my colleagues that an abscess had developed in the brain under this burn. I trephined through what appeared to be the dead bone, made search for an abscess, and found about a tablespoonful of pus which welled up through the wound. Two days after, the boy was able to move his foot and hand ; he made a rapid recovery and left the hospital with the use of his arm and leg.

The other case is that of a hod carrier, a young colored man, who fell thirty-eight feet. Those who saw him fall say he struck with his back against a barrel. He was entirely unconscious, and remained so for about an hour; then he recovered his senses, but he had no use of his right arm or leg. That is the history of the case. This accident occurred on the 5th day of December, 1888, nearly a year ago. He remained in that condition until brought to the West Penn. Hospital, in May. His pupils were normal, his tongue straight, and he answered questions rationally. His functions were all properly performed, except that he had no use of his right arm and leg ; sensation and motion were destroyed. Search was made for evidence of a fracture, but none could be found ; search was made for lesions of the scalp, but none could be found, except that a little over the right eye there was the cicatrix of a wound. This was all. He was treated, as the records show, with mercurials, with iodide of potash, under the supposition that possibly syphilis had something to do with it, but without improvement.

With Dr. McKennan's assistance, I trephined before the class on the 28th of September. The man was brought into the operating room and examined before the students, and it was found he could move no part of his right arm. His right leg was examined with the same result. Not the smallest motion. No sensation could be felt in the arm until the breast was reached in front, and the scapula behind, and no sensation could be felt in the leg and thigh until we came up on the lumbar region. This paralysis of sensation and motion of the right arm and leg pointed to a lesion of the motor areas about the fissure of Rolando. The head was shaved, the position of the fissure of Rolando was determined, and with the assistance of Dr. McKennan, an opening was made with a large trephine, one and one-half inches in diameter, over the upper third of the fissure of Rolando, which is the guide to the centers of motion of the arm and leg. This paralysis having come on within an hour after the fall, I expected to find a depression of the inner table of the skull. I had no guide to trephine by excepting the symptoms ; there was no injury to the external part of the skull, no injury to the scalp. I removed this large button of bone and found no lesion of the inner table of the skull. Neither was there any evidence of an abscess between the skull and the dura, but this latter was tense, and when the finger was put upon it gave a sense of fluctuation below. I cut through the dura and there was an escape of quite a quantity of dark blood ; the dura adherent to the arachnoid below and the membranes all glued together. There was only this escape of bloody fluid at the upper third of the fissure. The hemorrhage was easily stopped, and then it was found that the substance of the brain was in an almost fluid condition ; it was soft ; so much so that putting my finger into the aperture to feel, a little of the brain substance escaped through the wound. That was all I found. I confess that I was greatly disappointed. The dura was stitched with cat-gut, a drainage tube was inserted at the upper part of the wound, and it was dressed antiseptically. The patient almost expired on the table. He was taken to bed suffering greatly from exhaustion, but under the use of stimulants he revived, and on the next day, within twenty-four hours after the operation, he pulled up his right leg and kicked vigorously, the leg that had been dead for ten months. From that time he went on gradually improving, sensation was at once restored in the leg and in the right arm, but in the latter there was no motion. In three weeks from that time, the man walked into the operating room and showed himself to the class; I have him here to-night and want to show him to you. Sensation has been restored to the right arm, but motion of the hand is not quite perfect yet. The wound healed entirely without suppuration. I should say there were some manifestations of nervous force exhibited during his convalescence; a short time after the operation he was seized with a convulsion of the whole body. He was talking to his companion in the adjoining bed, and all at once the power of speech left him, and for half an hour he could not articulate a syllable. These are the facts in the case. I do not know why he is better. I can only say that probably some pressure was relieved, some pressure on a portion of the brain relieved by removing this large circle of bone. Why it is so I am not able to say. Like the man born blind in the Scripture, who only knew that whereas he was blind, now he could see, so I only know that though this man had the use of neither his right leg nor his right arm, now he can use both.

DR. BUCHANAN.—I saw this case on the third day, when the patient was at his worst. I believe he had a convulsion that day, and was inclined to be comatose when I saw him. It seemed to me at the time that it would have been the best thing to re-open that wound and discover whether there was not fluid beneath which was giving rise to the symptoms of compression. It may have been fortunate for the man that nothing of the kind was done, as whatever was giving rise to the trouble was absorbed. However, I think it would have been a perfectly safe procedure, with proper precautions, to have re-opened the wound and made a slight exploration to discover whether there was any clot or product of inflammation which might give rise to these symptoms of compression. I think the case a very remarkable one, and hardly anything can be added to what Dr. Murdoch has said.

DR. MCKENNAN.—When the man received the injury, according to the spectators, he fell upon his back across a barrel. We know that there might have been injury to the spinal cord, but there were no such symptoms. During the time the man was under the anæsthetic, there was a peculiarity that I noticed which Dr. Murdoch did not speak of, and that was during the latter stage of the excitement due to the anæsthetic, there was contraction of the right arm and leg. I have never read of anything of the kind, and the case is certainly remarkable.

DR. MCCANN.—I have been to some extent familiar with the case, as I saw the man frequently. The result of the operation has been exceedingly gratifying. Pressure was taken off the brain by the removal of this large button of bone, a quantity of blood, perhaps a drop of pus, was allowed to escape, and in addition to this a portion of the disintegrated brain tissue. Then drainage was provided, although the discharge was not great.

It is an example of what can be accomplished in apparently hopeless cases. It is only five years since any deliberate and well defined steps have been taken with regard to surgery of the brain. Before that time, everything that was done was groping in the dark. Macewen did the first operation in Glasgow nearly a dozen years ago. This case, I think, was not reported, or at least not reported for a considerable time afterward. Now, within the past few years, dealing with the brain has been entirely revolutionized. There is danger, of course, that there may be too much brain surgery, just as there is danger of too much surgery in other portions of the body; nevertheless the fact remains that operations on the brain to-day can be done with great safety, and are attended by beneficial results. Two other cases have also been operated on in the hospital recently. One, a Scotchman, who had received a fracture of the frontal bone, and who, as a result, was affected by movements of his head. His head was violently drawn to one side, so that you would suppose he would break his neck in the convulsive movements.

This seemed to result from the accident he had met with. There was a small cicatrix upon the frontal bone on the left side, which was trephined by Dr. King, one of my colleagues. Nothing was found underneath except an adherent dura, and underneath it a cyst, rather small, probably the result of a hemorrhage which had occurred at the time of the accident. In this instance, it seemed that little had been accomplished, as if the operation had done no good. However, the result of the operation was that the man was cured entirely and left the hospital well. In the other instance, a boy was struck on the head several months before with a missile. There were no well marked symptoms, no distinct paralysis. His condition, however, was gradually becoming worse, and with the consent and advice of my colleagues, I trephined him over what seemed to be the seat of the injury. After cutting out the button of bone, just as in Dr. King's case, little was found, nothing but an adherent dura.

Following this operation, which apparently was unproductive at the time, the boy regained his reason, and so far as I know, has continued well ever since. He remained in the hospital for a month afterward and felt able to resume his vocation. I think Dr. Murdoch's case was an interesting and instructive one, likely to lead to beneficial results. I am aware that there is danger of carrying this too far, that there is danger of transforming the man from a paralytic to an idiot by carrying the incisions too far, or by attempting too much with these very delicate structures; nevertheless I believe that within certain limits surgery of the brain is very efficacious, and that it is destined to accomplish a great deal of good for the human race.

DR. MUNN.—Dr. McCann has referred to the history of cerebral surgery and to some of the first operations as having been done while the operators were groping in the dark. In order that we may give honor to whom honor is due, it may be as well to definitely state the first operation. In the year 1876, Dr. Wm. Macewen, of Glasgow, was called to attend a boy who had been struck on the head and in whose case there developed some months later symptoms which indicated a lesion of the brain. He proposed an operation not at the seat of the injury but over the fissure of Rolando. Consent was refused, the boy died twenty-hours later and he was given permission to operate as he would have done prior to death. Removing the button of bone, nothing was seen on the surface, but plunging the scalpel into the brain substance an abscess was opened.

In 1883, Dr. Macewen performed the first operation of the kind that is on record. I might mention the history of it although it is something similar to Dr. Murdoch's case, with this exception, that the woman on whom the operation was performed was no doubt syphilitic. Mercury and iodide had failed to relieve her, and paralysis of one of the lower limbs had set in. He trephined over the upper portion of the ascending parietal convolution and from that locality evacuated a quantity of serous fluid. The woman made a recovery and had perfect health for some years. This case was reported in the early part of 1884 before the Glasgow Pathological Society, and a note of the case was published in the Glasgow *Medical Journal.* In 1885, Dr. Ferrier, of London, heard what had been done and went north to see what Macewen was doing in brain surgery. He returned to London and six weeks later assisted Messrs. Bennett and Godlee to perform their first operation. No doubt the credit of the first operation in surgery of the brain belongs to that Scotchman in the city of Glasgow.

### TINCTURE OF IODINE IN MIDDLE EAR DISEASE.

DR. ALLYN.—I would like to call attention to the use of tincture of iodine in the treatment of a species of chronic middle ear disease. To arrest discharge from an ear suffering for any length of time, I have many times been unable, with the use of all applications usually made, powdered iodoform, bichloride, and all the others I could bring into use. Many times the cause of this is the presence in the ear of a substance, whitish, tough, of an aspergillus nature. It has the faculty of reproducing in one night all that you can remove during the previous day's work. It adheres to the skin and grows very rapidly. I have been embarrassed with several of these cases, trying nitrate of silver up to the 10th per cent. and 20th per cent., applying it to the ears after I had tested the power to bear such strong applications. Under a two dram solution this seemed to thrive. I know the danger of using iodine in the middle ear. A case came to me that had been treated before, and all the applications of bichloride and of nitrate of silver in the strongest degrees failed to make any impression. I mopped the ear in the inner parts with vaseline, protecting it as thoroughly as possible. Then I made a solution of glycerine and tincture of iodine and worked upon the parts exposed, touching only the parts visible.

I succeeded in clearing that ear perfectly, leaving not the slightest particle of pus. I did that in two weeks, seeing the patient each day. Another case which had been running through childhood, I succeeded, a few weeks ago, in stopping the discharge for about a month only. After trying all the other applications which I felt were safe, I resorted to the iodine treatment. After mopping with vaseline, I first introduced glycerine into the ear, with no bad results. I then added about a tenth part of the tincture of iodine to it without bad results.

After the operation was made, I filled the ear thoroughly with clear water, followed it with iodoform and it was the last application I needed to make.

NASAL DIPHTHERIA.

DR. GREEN.—About four weeks ago I was called to see two cases of diphtheria. The family had lost two children, one of them just one week previous. The child had nasal diphtheria; the membranes had grown down so as to be distinctly perceptible. Small membranes on both tonsils. I gave large doses of calomel frequently administered, and made a tampon of absorbent cotton wet with a solution of bichloride of mercury one to a thousand, and plugged the nose as completely as I could. I allowed it to remain twenty minutes. Before doing this I syringed the nose thoroughly with a solution of borax. I left some of the mercury solution, and told the mother to place the cotton every two hours until my return. The same evening I applied iodoform, reduced with calcined magnesia. On my visit the next day at ten o'clock the membranes had entirely disappeared from the nose. Hemorrhages occurred infrequently, but there was no reappearance of the membranes. About one week from that time I saw a similar case in a little girl about five and a half years of age. I subjected the patient to the same treatment, and the case rapidly recovered.

DR. HUSELTON.—Did the membranes from the throat disappear at the same time with the membranes of the nose?

DR. GREEN.—They disappeared fully within forty-eight hours. I gave large doses of calomel, ten grains per hour.

DR. DAVIS.—I would like to ask Dr. Green for how many hours he kept that up.

DR. GREEN.—I used mild chloride of mercury in large doses frequently administered. I administered to the first patient I think about 120 grains in the first twenty-four hours. The membranes from the throat disappeared not as rapidly as the membranes from the nose.

DR. LANGE.—I desire to say that I think Dr. Green's treatment very judicious, especially the ten grains of calomel per hour. I myself give infants five grains of calomel every hour for three or four hours without salivation, without purgation. This has been so universally my experience, that calomel does not purge in diphtheria, that I sometimes take this as a criterion as to whether I have a case of diphtheria or an aggravated case of follicular tonsillitis. In such cases where the calomel has purged the patient I have concluded that I had not to do with diphtheria, because of my universal experience that in diphtheria calomel in ten-grain doses does not purge nor salivate as a rule. I remember one boy who had nasal diphtheria who received at my hands one ounce and a scruple of calomel during ten days and was not purged, not ptyalized, and who recovered.

DR. THOMAS.—I would like to state my experience of the calomel treatment of diphtheria. Every case that was malignant and that was at all aggravated died without exception. That is the history of what I saw of this practice with the large doses of calomel.

DR. KŒNIG.—For my part I desire to commend Dr. Green for the other treatment, perhaps also for the calomel treatment. I think at the present day no one denies the value of antiseptics in diphtheria, and every one will admit that all of the remedies, with the exception perhaps of some that are recognized at the present time as of no virtue in diphtheria, are antiseptics. Calomel itself, I imagine, is beneficial, because of its antiseptic properties. No doubt when these large doses of calomel are administered more or less of it becomes entangled in the meshes of the membrane. I see no reason why it should not exert its antiseptic properties.

DR. McCANN.—I want to say a little about calomel treatment in diphtheria. I have seen it, have been using it for twenty-five years. I have administered five grains per hour to a child eight months old, and I have done this, not once, but repeatedly, and I have certainly seen most marvellous results follow this treatment. As malignant cases of diphtheria as I have ever seen recovered under the treatment. Now I have not seen bad results follow the administration of calomel. In fact, the plan of treatment which, in my experience, has been successful has been the calomel treatment. I have seen it in my own family, have tried it among my own children when they were in a condition where death seemed to be impending. I have seen it tried in other families where, under other plans of treatment, the children died; and what is the plan of treatment to-day outside of the mercurial treatment? The treatment which was advocated thirty years ago—iron and chlorate of potash. This is the remedy which the profession has to offer against the mercurial treatment.

DR. HUSELTON.—I to-day use bichloride of mercury. This can be put up in such shape that it is very pleasant to take.

